# Breathlessness

**Published:** 1905-06-10

**Authors:** 


### BREATHLESSNESS.

Sir Lauder Brunton, in an interesting review[1] of some features of breathlessness, points out that for the proper aeration of the blood two factors are essential: firstly, that fresh air shall freely enter the lungs; and, secondly, that the blood shall flow through them in sufficient quantity, interference with either of which processes will produce breathlessness. But breathlessness includes both excessive breathing without distress and excessive breathing with distress, the latter representing what is to be understood by dyspnœa, the former being exemplified by the hurried respirations which attend exertion in states of perfect health. Dyspnœa itself is composed of two elements, one central and one peripheral, and the central impression of breathlessness may be out of all proportion to the peripheral cause in the heart or lungs, a condition seen in some hysterical states and also in chronic heart disease. Excluding gross lesions of the lungs and pleuræ, the prime cause of dyspnœa is muscular inefficiency of the heart for the work before it, whether this arise from faulty nutrition of its muscle as in cases of coronary sclerosis (the work required remaining the same), or from increase in its task through valvular deficiencies (the nutrition of its fibres remaining unimpaired). Thus aortic valvular disease is not marked by dyspnœa, because the pulmonary circulation is not disturbed so long as the mitral valve maintains its functional integrity, but when this becomes incompetent, or worse still, permanently narrowed, then the pulmonary circuit becomes obstructed and dyspnœa results.

The great object in the treatment of cardiac dyspnœa is therefore to facilitate the pulmonary circulation, and the most important means is rest. Every muscular movement tends to accelerate respiration, and when the heart is already working to the full of its capabilities, any movement, even the most insignificant, exaggerates the difficulties of the already overtaxed heart-muscle. Therefore the wise maxim for such patients is "Do nothing that any one else can do for you," and rest must be absolute, either in bed, or, in the case of orthopnœa, in a chair. Next in importance comes massage, for it assists the return of blood to the heart, and in this way diminishes the labours of this organ. "When actual dyspnœa is present, movements, however slight, are to be avoided, but when the heart begins to recover and the patient is able for it, graduated movements and the Nauheim baths are most useful in aiding recovery."

Sir Lauder Brunton shares the common view that no drug is so useful in these cases as digitalis, and recommends its administration in the form of a pill composed as follows:—Powdered digitalis, one grain; powdered squill, one grain; and blue pill, one grain; free purgation also is an invaluable help in the relief of dyspnœa. The author makes a strong pronouncement in favour of opium. "For some reason or another there is quite an unnecessary fear both of this drug and of mercury . . . it was with reference to this drug that I tried to emphasise so much the distinction between the peripheral cause and the cerebral sensation of dyspnœa. In many cases of cardiac disease the patient is prevented from falling asleep by a sudden start or a suffocative feeling, and his condition is rendered materially worse by the exhaustion consequent upon want of sleep. In cardiac dyspnœa there is no drug which will give such immediate relief as opium, and it may be administered either by the mouth, or as a subcutaneous injection of morphine, or by mixing from half a drachm to a drachm of the tincture with one or two drachms of water, and injecting this amount into the rectum with an ordinary glycerine syringe." In conclusion, Sir Lauder lays particular stress on the importance of gastric distension by gas as a cause of dyspnœa in cardiac cases, and recommends for the abatement of the condition a milk diet and the use of carminatives.

[1] Pract., June 1905.

